# Effect of Plyometric Jump Training on Vertical Jump Indicators and Performance-Related General Physical Fitness in Rugby Players: A Systematic Review

**DOI:** 10.3390/life16050859

**Published:** 2026-05-21

**Authors:** Javier Russell-Guzmán, Sebastián Moraga-Moraga, Alexis Espinoza-Salinas, Felipe Inostroza-Ríos, Claudio Carvajal-Parodi, Francisco Guede-Rojas, David Ulloa-Díaz, Jorge Pérez-Contreras

**Affiliations:** 1 Escuela de Kinesiología, Universidad Santo Tomás, Santiago 8320000, Chile; jrussellguzman@santotomas.cl (J.R.-G.); alexisespinozasa@satotomas.cl (A.E.-S.); 2 Carrera de Ciencias de la Actividad Física y el Deporte, Universidad SEK Chile, Santiago 7500000, Chile; 3 Facultad de Salud y Ciencias Sociales, Universidad de las Américas, Santiago 8320000, Chile; sebamoraga.91@gmail.com; 4 Post-Graduate Program of Physical Education, Federal University of Juiz de Fora, Governador Valadares 35010-180, Brazil; felipe.inostroza.311@gmail.com; 5 Escuela de Kinesiología, Facultad de Ciencias de la Rehabilitación y Calidad de Vida, Universidad San Sebastián, Concepción 4030000, Chile; claudio.carvajal@uss.cl; 6 Exercise and Rehabilitation Science Institute, School of Physical Therapy, Faculty of Rehabilitation Science, Universidad Andres Bello, Santiago 7591538, Chile; francisco.guede@unab.cl; 7 Department of Sports Sciences and Physical Conditioning, Universidad Católica de la Santísima Concepción, Concepción 4030000, Chile; 8 Magíster en Evaluación y Planificación del Entrenamiento Deportivo, Facultad de Ciencias de la Vida, Universidad Viña del Mar, Valparaíso 2520000, Chile; 9 Departamento de Educación Física, Deportes y Recreación, Universidad Metropolitana de Ciencias de la Educación, Santiago 7500000, Chile

**Keywords:** plyometrics, complex training, vertical jump, physical fitness, rugby, performance

## Abstract

Introduction: Vertical jump performance is linked to key performance indicators in rugby, including tackling success and ruck involvement. Although plyometric jump training (PJT) is known to enhance explosive qualities in various sports, its specific effects in rugby remain unclear. Objective: To synthesise evidence on the effects of PJT on vertical jump ability and other physical fitness components in adult rugby players. Methods: A systematic review was conducted in accordance with PRISMA 2020 guidelines. PubMed, EBSCO (SPORTDiscus), WoS, and Scopus were searched up to December 2025. Experimental and quasi-experimental studies involving rugby players undertaking PJT programmes of at least two weeks, with at least one vertical jump outcome, were included. Two reviewers independently performed study selection and data extraction. Risk of bias was assessed using the RoB 2.0 tool. Results: Seven studies involving 178 male players were included. PJT improved sprint speed, change of direction, anaerobic power, reactive strength, lower-limb stiffness, and isometric plantar flexion strength. Gains in countermovement jump power were noted in some conditions, such as training on softer surfaces. However, improvements in jump height were inconsistent. Conclusion: PJT enhances several important physical qualities in rugby players but shows variable effects on vertical jump height. Further high-quality research is needed.

## 1. Introduction

Rugby is a team contact sport that has achieved high global popularity [1]. The literature has identified key factors associated with rugby performance, highlighting technical-tactical indicators such as the number of tries scored, the percentage of successful tackles, and effective participation in offensive and defensive rucks [2]. These indicators show a clear association with various physical fitness manifestations, particularly cardiorespiratory capacity, speed, strength, and vertical jump performance [2]. Notably, vertical jump variables exhibit significant correlations with multiple sports performance indicators regardless of playing position, suggesting that improving this capacity could translate into direct benefits.

Plyometric jump training (PJT) emerges as a promising methodology for developing these capacities, offering comparative advantages over traditional methods by producing neuromuscular adaptations specific to explosive actions such as jumping, acceleration, and change of direction [3]. It is noteworthy that plyometrics are frequently integrated into complex or contrast training schemes, where actions are introduced following the performance of high-intensity strength exercises (≈85% 1 RM) to effectively activate post-activation potentiation (PAP); nonetheless, some research suggests that moderate loads near 65% 1 RM could also generate beneficial effects in certain contexts and populations [4,5]. This strategy has demonstrated its efficacy across multiple sporting disciplines, including basketball [6,7] and soccer [8,9,10].

Despite the evidence linking vertical jump performance to competitive success in rugby and the documented positive effects of plyometric training in other sports, the specific effects of plyometric training on rugby players remain unknown. Therefore, the aim of this review was to synthesize the available evidence regarding the effects of plyometric jump training on vertical jump capacity and other performance-related physical fitness components in adult rugby players. The findings may help elucidate the effects of plyometric training while establishing evidence-based guidelines to optimize training prescriptions and contribute to the development of a specific theoretical framework for this sport.

## 2. Materials and Methods

The present study adhered to the protocols established by the Preferred Reporting Items for Systematic Reviews and Meta-Analyses (PRISMA) 2020 statement [11]. The review protocol was approved by the Ethics Committee of the Universidad Santo Tomás (CEC 142.24) and was registered on the International Prospective Register of Systematic Reviews (PROSPERO) website on 27 February 2026 (CRD420261327392).

### 2.1. Selection Criteria

The PICOT methodology was used to refine the research question and the information search. Furthermore, the inclusion and exclusion criteria for article selection are presented in Table 1.

### 2.2. Electronic Search Strategy

The search was conducted by two researchers (J.R-G.; S.M-M.) by consulting the available literature up to December 31, 2024. The PubMed, Web of Science, Scopus, and EBSCOhost (SPORTDiscus) platforms were consulted. The search strategy was constructed by combining controlled descriptors from the respective thesauri (MeSH) and relevant free-text terms for the population, intervention, and outcomes of interest. The strategy was optimized for each database using the Boolean operators “AND” and “OR” as appropriate. The search strategies for each database are described in Table 2.

### 2.3. Study Selection Eligibility

Total search results were exported from the databases in RIS or CSV format and subsequently managed using the Rayyan platform [12]. Two reviewers (J.R-G.; S.M-M.) performed the process independently; in cases where consensus was not reached, a third reviewer (J.P-C.) was consulted. Duplicates were removed, followed by an initial screening by title and abstract. Finally, the full text was analysed to ensure compliance with the selection criteria.

### 2.4. Data Collection Process

The selected articles were compiled and synthesized into a summary table by three researchers (J.R-G.; S.M-M.; F.I-R.). This table includes the primary author’s name, sample characteristics (sample size, mean age, sporting experience, etc.), characteristics of the training protocol or intervention (weekly frequency, intensity, time, exercise modality, total duration, and progression), and results obtained for the variables of interest. A quantitative meta-analysis was not performed due to substantial methodological heterogeneity among studies, including differences in plyometric protocols, intervention duration, outcome measures, testing procedures, and incomplete reporting of variance data required for pooled effect size calculations. A sensitivity approach was used, assessing the consistency of findings in studies with different methodological characteristics and levels of risk of bias.

### 2.5. Risk of Bias Assessment

The risk of bias of the selected results was analysed using the RoB 2.0 tool [13]. This analysis focuses on five key domains: bias arising from the randomization process, bias due to deviations from intended interventions, bias due to missing outcome data, bias in measurement of the outcome, and bias in selection of the reported result. Two independent reviewers (J.R-G.; S.M-M.) conducted the assessment, using a series of criteria to classify the risk in each domain as high, low, or of some concerns; when consensus was not reached, a third reviewer (J.C-P.) was consulted. The criteria are detailed in Table 3. Discrepancies between evaluators were resolved through discussion or, if necessary, through third-party mediation.

## 3. Results

### 3.1. Search Results

**Figure 1:**
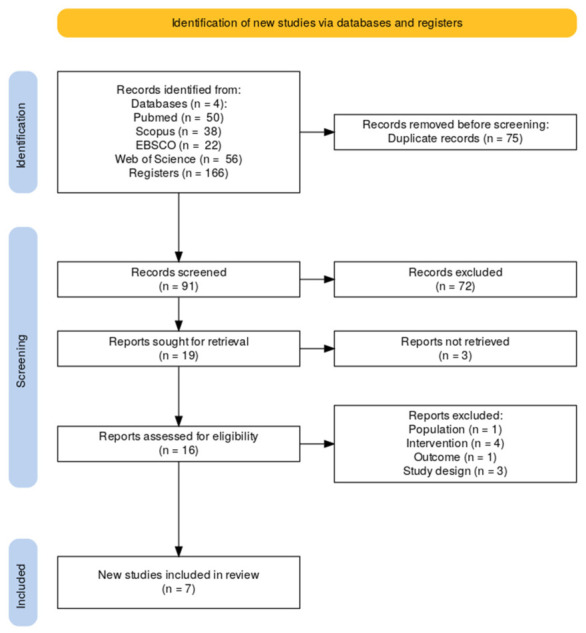
Flow diagram created using the PRISMA 2020 flow diagram template [15].

The initial search identified 166 records. After removing 75 duplicates, 91 articles were retained for the screening phase. During the title and abstract evaluation, 72 records were excluded, resulting in 19 articles eligible for full-text assessment. Of these, 3 could not be retrieved in full-text; therefore, 16 were evaluated in depth. Following this assessment, 9 studies were excluded for not meeting the eligibility criteria. Consequently, a total of 7 studies were included for the qualitative synthetic analysis. The flow diagram of this process is presented in Figure 1.

### 3.2. Study Characteristics and Risk of Bias

**Figure 2:**
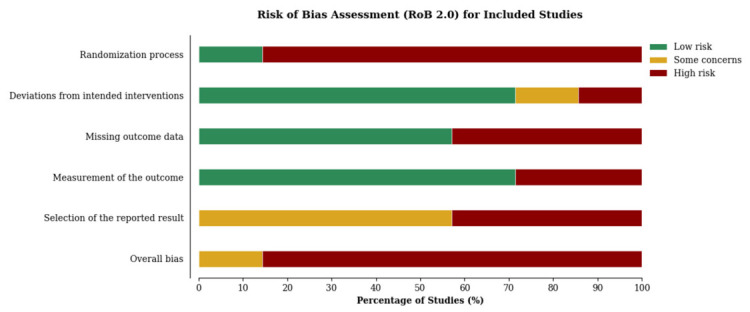
Distribution of the risk of bias identified in the included studies.

**Figure 3:**
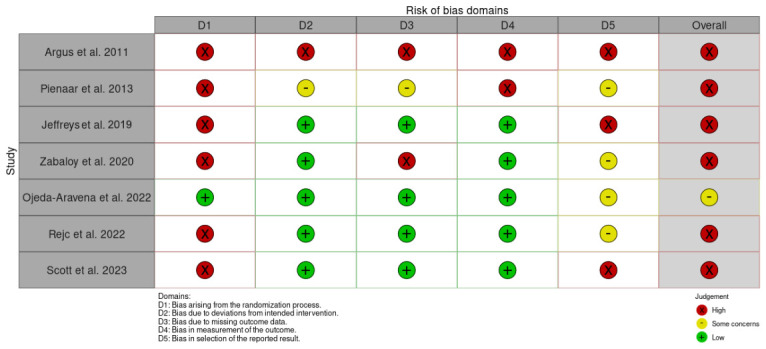
Identification of the risk of bias by domains in the included studies [16,17,18,19,20,21,22].

The risk of bias analysis revealed limited overall methodological reliability in the available literature. All included studies adopted a “per-protocol” analysis. The assessment results are detailed in Figure 2 and Figure 3. The most critical domain was the randomization process, where the vast majority of studies (85.7%) presented a high risk of bias. Only one study (14.3%) implemented an adequate randomization sequence. Conversely, the domain of deviations from intended interventions showed a generally low risk (71.4% of studies with low risk). However, significant deficiencies were identified in the measurement of outcomes (28.6% with high risk) and, especially, in the management of missing data (42.9% with high risk) and the selection of reported results (42.9% with high risk and 57.1% with some concerns). Consequently, the overall assessment classified six studies (85.7%) with a high overall risk of bias. Only the research by Ojeda-Aravena et al. [16] raised some concerns regarding overall risk, being the only one not categorized as high risk. Therefore, the available evidence should be interpreted with caution, as the predominance of studies with high risk of bias limits the certainty and generalizability of the observed findings.

The methodological and population characteristics of the included studies are summarized in Table 4. The total combined sample comprised 178 participants, exclusively male, with an average age range between 18.9 and 24 years. Regarding competitive level, the study population included professional athletes [16,17,18], senior players [19] and university players [20,21,22].

Regarding the plyometric training modality implemented, only 28.6% of the studies [22,23] employed a complex training modality that combined plyometric exercises followed by strength exercises within the same training session. The majority of the interventions, 57.1% [18,19,20,21], corresponded to programs in which plyometric blocks were implemented independently, integrated within a general planning that also included strength training sessions. It is important to specify that these combined training interventions did not follow a complex or contrast training model, but rather the coexistence of both stimuli within the same physical preparation program. Finally, one study, 14.3% [16], investigated the effects of plyometric training implemented on different surfaces (stiff versus soft).

The duration of the plyometric interventions ranged from 4 to 8 weeks, with the 4-week period being the most frequent [16,17,21]. Only 57.1% of the studies [16,17,18,19] reported session duration in minutes. Regarding training frequency, this varied between 2 and 3 weekly sessions. Most studies (71.4%) implemented a frequency of 2 sessions per week [17,18,19,20,22], while the remaining 28.6% [16,21] employed a frequency of 3 sessions per week. 

The included studies reported that plyometric training yields significant improvements in various performance variables. Specifically, improvements were observed in 20 m sprint performance, change of direction, and anaerobic power [21]. Furthermore, plyometric training demonstrated significant improvements in the reactive strength index (RSI) and lower-body stiffness [20], as well as in isometric plantar flexion strength [18]. Increases in countermovement jump (CMJ) power were also recorded in the group that trained on a soft surface when comparing pre- and post-intervention measurements. However, due to the methodological heterogeneity and the elevated risk of bias identified across the included studies, these findings should be interpreted as exploratory associations rather than definitive evidence of effectiveness.

It is noteworthy that most studies did not report significant positive effects on vertical jump height, with the exception of the group that performed plyometric training on a soft surface, which showed improvements in CMJ height [16]. This finding suggests that the practical benefits of plyometric jump training in rugby may be more consistently related to reactive strength, sprint ability, and force-production variables than to vertical jump height itself. A summary of the studies is shown in Table 5.

## 4. Discussion

This systematic review examined the effects of plyometric jump training on vertical jump variables and physical fitness components associated with performance in rugby players. The findings suggest that this training modality may be associated with improvements in muscular power, linear sprint speed, change of direction, muscle stiffness, lower-body reactive strength, and isometric plantar flexion strength. However, no consistent effects were observed on vertical jump height, except for interventions performed on soft surfaces. Considering the methodological limitations and heterogeneity of the included studies, the present findings should be interpreted cautiously and as indicative of potential rather than definitive performance benefits.

Rugby is a high-intensity intermittent discipline [24] that demands optimal development of physical components such as strength, power, and speed [23,25]. Plyometric training may represent a useful methodological strategy to develop these capacities [26]. From a physiological perspective, this training modality optimizes force production within brief time intervals by utilizing the elastic components of the muscle–tendon complex and enhancing the reflex mechanisms of the stretch-shortening cycle [27]. These neuromuscular adaptations favour the execution of explosive actions characteristic of rugby, such as accelerations, changes of direction, and jumps [3]. Current evidence suggests that plyometric training, either as a standalone modality or integrated into complex programs, may contribute to improvements in physical performance indicators across various sports disciplines [6,7,8,9,10,28].

Among the included studies, only three evaluated the effect of plyometric training on vertical jump height using CMJ or SJ tests [16,17,19], with significant improvements observed only in the study by Ojeda-Aravena et al. [16] under specific soft-surface conditions and in the CMJ with arm swing modality. This limited effectiveness could be attributed to the short duration of the interventions (4–7 weeks), which is below the 10-week threshold suggested by other researchers to optimize neuromuscular adaptations [29]. Additionally, the greater improvement in CMJ with arm swing aligns with evidence reporting larger gains in actions involving a prolonged stretch-shortening cycle (SSC) [30].

Regarding reactive strength, assessed in two studies using drop jumps (DJ) from 30–60 cm [20,22], significant improvements were observed with low volumes (36–40 jumps/week) in 6-week programs. However, a recent meta-analysis suggests that longer interventions (>7 weeks) and a higher number of sessions (>14) may optimize these adaptations [31]. This indicates that rugby programs may be underutilizing the potential of plyometric training by employing sub-optimal durations. These findings should be interpreted cautiously given the methodological heterogeneity and elevated risk of bias identified across the included studies.

In terms of power and rate of force development, three studies reported improvements in CMJ [16,18,22] and in the concentric phase of the jump [16]. These findings support the specificity of the prolonged SSC for enhancing the ability to produce force rapidly [3], which is crucial for explosive actions in rugby. These findings should be interpreted cautiously given the methodological heterogeneity and elevated risk of bias identified across the included studies.

The practical relevance of these adaptations is supported by reported correlations between DJ and CMJ performance and rugby performance indicators, such as dominant collisions, line breaks, and tries scored [2]. While the observational nature of these studies limits causal inferences, the consistency of the findings suggests that plyometric training could positively transfer to competitive performance. Although standardized effect sizes were inconsistently reported across studies, the percentage improvements described in sprint and reactive strength outcomes may be considered practically meaningful in rugby contexts where small performance gains can influence match actions and competitive success.

Regarding other physical fitness components, only one study evaluated the effect of plyometric training on changes of direction, reporting a significant improvement of 2.8% [21]. This finding coincides with previous systematic reviews in other team sports [32] and is particularly relevant for rugby, where changes of direction directly impact decisive actions such as offensive penetrations and tackling effectiveness [33]. These findings should be interpreted cautiously given the methodological heterogeneity and elevated risk of bias identified across the included studies.

Three studies documented improvements in sprint performance over distances between 5–30 m [19,21,22], aligning with existing evidence on the effects of plyometric training on acceleration [3,34]. These improvements are crucial for rugby, where sprint performance correlates with indicators such as carries and offside line breaks in forwards [2]. Notably, none of the studies evaluated kinetic variables such as linear momentum (the product of mass × velocity), which has been associated with ball possession, dominant collisions in backs, and passing efficacy [2]. Given the high body mass characteristic of rugby players, improvements in speed through plyometric training could significantly enhance physical impact during match play. These findings should be interpreted cautiously given the methodological heterogeneity and elevated risk of bias identified across the included studies.

Four studies analysed strength and power indicators not specific to vertical jumping [18,19,21,22]. Improvements were reported in (1) anaerobic power in the 30 s Wingate test [21], a parameter relevant for backs given the anaerobic predominance of their efforts [35]; (2) isometric plantar flexion strength [18]; and (3) maximum squat strength [22]. Nevertheless, these results should be interpreted with caution due to methodological heterogeneity: the study by Rejc et al. [18] utilized specialized ergometric equipment, while Scott et al. [22] observed improvements only under variable load conditions with elastic implementation. These findings should be interpreted cautiously given the methodological heterogeneity and elevated risk of bias identified across the included studies.

Despite the fact that vertical jump height did not improve consistently, improvements were observed in indicators with greater transfer to rugby (sprint, change of direction, reactive strength). This suggests that the value of plyometric training in rugby may lie more in its ability to enhance sport-specific skills than in improving vertical jump height itself. These findings should be interpreted cautiously given the methodological heterogeneity and elevated risk of bias identified across the included studies.

A critical finding of this systematic review was the high risk of bias identified in the majority of the included studies, particularly in the domain related to randomization processes. The most poorly reported aspect was allocation sequence concealment, a fundamental element for minimizing selection bias [14]. The presence of this bias substantially compromises the internal validity of the findings, potentially leading to inaccurate estimates of the true effect size of the interventions. Additionally, none of the studies met the criteria for low risk of bias in the domain of selection of the reported result, suggesting possible selective reporting practices. The implementation of robust randomization protocols and prospective study registration emerge as indispensable methodological requirements to strengthen the credibility of future evidence in this area. This methodological limitation reinforces the exploratory nature of the conclusions and highlights the need for future randomized controlled trials with improved reporting standards and larger sample sizes. Overall certainty in the available evidence remains limited due to the predominance of studies with high risk of bias and heterogeneous intervention protocols.

## 5. Conclusions

The findings of this systematic review suggest that plyometric jump training programs may be associated with potentially beneficial neuromuscular adaptations for sub-elite male rugby players. In particular, the evidence suggests that interventions of 4–8 weeks in duration, with a frequency of 2–3 weekly sessions—applied either as complementary plyometric sessions to strength training or integrated into a complex training modality within the same session—are associated with consistent improvements in (1) mechanical power during explosive actions, (2) performance in short linear sprints (5–20 m), (3) change of direction ability, and (4) muscle–tendon stiffness and lower-body reactive strength. However, critical limitations were identified that condition the robustness of these conclusions. The predominant high risk of bias, along with the methodological heterogeneity in the intervention protocols, necessitates a cautious interpretation of these findings. Despite these limitations, the directional consistency of the observed effects suggests that plyometric training may represent a promising complementary strategy for developing physical qualities relevant to rugby performance. Plyometric jump training appears to be associated with potential improvements in reactive strength, sprint performance, and force-related capacities in rugby players; however, the evidence base remains limited by methodological heterogeneity and high risk of bias. Furthermore, improvements in vertical jump height were not consistently observed across the included studies. Although plyometric training has been incorporated into rugby conditioning since the 1990s, eligible studies were identified only from 2011 onwards. This may reflect a limited availability of rugby-specific experimental research and methodological constraints in earlier studies, highlighting an important gap in the longitudinal development of evidence in this field.

Future research should address current methodological gaps through designs with adequate statistical power and low risk of bias, precise description of prescription components (intensity, volume, density), inclusion of female populations, and evaluation of position-specific effects.

### 5.1. Limitations

The present review is not without limitations that should be considered when interpreting its findings. First, the exclusive inclusion of male populations limits the generalizability of the results to female rugby players, reflecting an existing gender gap in the scientific literature [10]. Second, insufficient reporting of key training prescription components was identified, particularly regarding intensity and progression, which hinders the replicability of the interventions. Third, methodological heterogeneity was considerable, manifested in the absence of control groups in 42.9% of the studies and the diversity of intervention protocols (isolated vs. combined plyometrics, different surfaces, and variable loading strategies). Finally, the high overall risk of bias observed in most of the included studies necessitates a cautious interpretation of the results.

### 5.2. Future Projections

Based on the identified findings and limitations, it is recommended that future research (1) deliberately include female populations to address the current gender gap; (2) meticulously document all training prescription components (intensity, volume, density, progression) to allow for replicability and meta-analysis; (3) adopt methodologically rigorous designs that minimize the risk of bias, with special attention to randomization, blinding, and prospective protocol registration; and (4) explore the dose–response relationship and the physiological mechanisms underlying plyometric training-induced adaptations in rugby players.

## Figures and Tables

**Table 1:** PICOT methodology, inclusion and exclusion criteria for search and results selection.

Research Question (PICOT)	
Population	Rugby players (union or league) of any competitive level (elite, sub-elite, amateur, youth) and gender.
Intervention	Plyometric training programs including jump exercises (e.g., depth jumps, countermovement jumps, hurdle jumps).
Comparison	Control groups that performed: (a) Usual/traditional training (e.g., strength, endurance training). (b) No additional training intervention.
Outcome	Primary: Variables derived from vertical jump tests: jump height, power output, force output, reactive strength index, and muscle stiffness. Secondary: Other physical performance measures not derived from jumping.
Study Design	Randomized controlled trials (RCTs), non-randomized controlled trials, and quasi-experimental studies.
Time:	Minimum intervention duration of 4 weeks for plyometric jump training programmers.
Eligibility Criteria	
Inclusion Criteria	(a) Experimental or quasi-experimental study design. (b) The study population consisted of rugby players. (c) The plyometric training intervention had a minimum duration of 4 weeks. (d) Articles retrieved in English or Spanish.
Exclusion Criteria	(a) Participants presented with declared musculoskeletal pathologies or injuries at the time of the study. (b) Studies did not report measurements of at least one primary outcome variable (vertical jump output). (c) Systematic reviews, meta-analyses, editorials, conference abstracts, and case studies were excluded.

**Table 2:** Search strategies by database.

By Database	Search Strategies
Pubmed	(“Rugby”[Mesh] OR “rugby”) AND (“plyometric exercise”[MeSH Terms] OR (“plyometric” AND “exercise”) OR “plyometric exercise” OR (“plyometric” AND “training”) OR “plyometric training”) AND (“performance” OR “fitness”)
Scopus	Rugby (Title-Abs-Key) AND plyometric (Title-Abs-Key) AND training (Title-Abs-Key) AND performance (Title-Abs-Key)
EBSCO (SPORTDiscus)	Rugby AND plyometric training AND performance
Web of Science	Rugby (Topic) AND Plyometric (Topic) AND training (Topic) AND performance (Topic)

**Table 3:** Risk of bias assessment criteria using RoB 2.0.

Domain	Low Risk	High Risk	Some Concerns
Randomization process	Adequately generated sequence and clearly described allocation concealment.	Non-random sequence, lack of concealment, or major imbalances in baseline characteristics.	Lack of information on methods used to generate the sequence or ensure concealment.
Deviations from intended interventions	Interventions implemented according to protocol, without influences from participants or non-blinded personnel.	Significant changes in interventions that could have affected results and are related to the assigned group.	Inconsistent supervision or lack of information on measures to mitigate influences from personnel or participants.
Missing outcome data	Low attrition rates distributed equally between groups, with appropriate handling of missing data.	High attrition rates, and missing data likely related to the outcomes or the intervention.	Moderate proportion of missing data or unclear data management.
Measurement of the outcome	Outcome assessors blinded to the assigned group, and results measured objectively with validated instruments.	Non-blinded assessors and/or subjective measurement of outcomes.	Lack of information on blinding or potential subjectivity in outcome measurement.
Selection of the reported result	All predefined outcomes were reported and a previously registered protocol was followed.	Only selected results are reported without justification, or reports do not match a registered protocol.	Lack of prior protocol registration or insufficient justification for the selection of reported results.

Adapted from the Cochrane Handbook [14].

**Table 4:** Methodological characteristics of the studies included in the systematic review.

Reference	Participants	Population Characteristics	Intervention Duration	Weekly Frequency	Training Modality
Argus et al. (2011) [17]	28	Male rugby players with 2 years of experience in strength and plyometric training	4 weeks	2 sessions	Complex training
Pienaar & Coetzee (2013) [21]	35	Male university players with 10–12 years of rugby experience	4 weeks	3 sessions	Combined training
Jeffreys et al. (2019) [20]	29	Male university players with 1–2 years of experience in plyometry	6 weeks	2 sessions	Combined training
Zabaloy et al. (2020) [19]	34	Male senior players with 10 years of rugby experience	7 weeks	2 sessions	Combined training
Ojeda-Aravena et al. (2022) [16]	14	Male players with 3 years of rugby experience	4 weeks	3 sessions	Combined training
Rejc et al. (2022) [18]	14	Elite male forwards (Italian national championship)	8 weeks	2 sessions	Combined training
Scott et al. (2023) [22]	24	Male university players with 6 months of strength training experience	6 weeks	2 sessions	Complex training

**Table 5:** Results of plyometric training interventions in rugby players.

Reference	Groups	Jump Test Performance Indicators	General Physical Performance Indicators
Argus et al. (2011) [17]	EG-F, EG-A, EG-R	CMJ: Height (%) ↔ EG-F (+1.3%); Height (%) ↔ EG-A (+6.7%); Height (%) ↔ EG-R (+4.0%)	Not reported
Pienaar & Coetzee (2013) [21]	EG	Sargent jump test: Height (cm) ↔ EG (+2.0%); Peak power (W) ↔ EG (+1.0%)	20 m Sprint (s) ↓ EG (−2.7%); *T*-test (s) ↓ EG (−2.8%). Wingate test (W): ↑ EG (+2.8%) *
Jeffreys et al. (2019) [20]	EG-LV, EG-HV	30 cm DJ: RSI ↑ EG-LV (+11.1%), RSI ↑ EG-HV (+5.7%). 45 cm DJ: RSI ↑ EG-LV (+9.0%), RSI ↑ EG-HV (+7.9%). 60 cm DJ: RSI ↑ EG-LV (+17.5%), RSI ↑ EG-HV (+9.6%). Multi-jumps (10 reps): Stiffness (kN·m^−1^) ↑ EG-LV (+2.4%), Stiffness (kN·m^−1^) ↑ EG-HV (+9.5%)	Not reported
Zabaloy et al. (2020) [19]	EG-NI, EG-VI, EG-SI, EG-B	SJ: Height (cm) ↔ EG-VI (+5.2%). CMJ: Height (cm) ↔ All groups	30 m Sprint (s) ↓ EG-SI (−1.2%), 30 m Sprint (s) ↓ EG-B (−1.4%). 1 RM Squat (kg) ↔ All groups
Ojeda-Aravena et al. (2022) [16]	EG-HS, EG-SS	SJ: Height (cm) ↔ EG-HS (+4.3%), Height (cm) ↔ EG-SS (+4.6%). CMJ: Height (cm) ↔ EG-HS (+5.2%), Height (cm) ↔ EG-SS (+1.6%). CMJ with arms: Height (cm) ↔ EG-HS (+5.3%), Height (cm) ↑ EG-SS (+8.2%)	Not reported
Rejc et al. (2022) [18]	EG	SJ: Peak power (W) ↔ EG (+0.8%). CMJ: Peak power (W) ↔ EG (−7.6%). DJ: Peak power (W) ↔ EG (−0.9%)	Max isometric knee extension strength (N) ↔ EG (+3.9%). Max isometric knee flexion strength (N) ↔ EG (+7.7%). Max isometric plantar flexion strength (N) ↑ EG (+35.9%) *
Scott et al. (2023) [22]	EG-VLC, EG-TLC	CMJ: Peak power (W) ↑ EG-VLC (+4.4%), Peak power (W) ↑ EG-TLC (+5.0%) *. 40 cm DJ: RSI ↑ EG-VLC (+20.4%), RSI ↔ EG-TLC (+20.7%). Multi-jumps (10 s): Stiffness (kN·m^−1^) ↑ EG-VLC (+6.3%), Stiffness (kN·m^−1^) ↔ EG-TLC (+3.3%)	1 RM Squat (kg) ↑ EG-VLC (+13.4%), 1 RM Squat (kg) ↑ EG-TLC (+16.0%). 5 m Sprint (s) ↓ EG-VLC (−5.6%), 5 m Sprint (s) ↓ EG-TLC (−8.1%). 20 m Sprint (s) ↔ EG-VLC (−1.6%), 20 m Sprint (s) ↓ EG-TLC (−3.3%) *

Abbreviations: EG: Experimental Group; EG-F: Experimental Group with Free jump; EG-A: Experimental Group with Assisted jump; EG-R: Experimental Group with Resisted jump; EG-LV: Experimental Group with Low Volume of plyometrics; EG-HV: Experimental Group with High Volume of plyometrics; EG-NI: Experimental Group with Non-Individualized loads; EG-VI: Experimental Group with load oriented to improve Velocity Imbalance; EG-SI: Experimental Group with load oriented to improve Strength Imbalance; EG-B: Experimental Group with Balanced subjects (strength-velocity); EG-HS: Experimental Group with plyometrics on Hard Surface; EG-SS: Experimental Group with plyometrics on Soft Surface; EG-VLC: Experimental Group with Variable Load Complex training; EG-TLC: Experimental Group with Traditional Load Complex training; SJ: Squat Jump; CMJ: Countermovement Jump; DJ: Depth Jump; RSI: Reactive Strength Index. Symbols: ↑: improvement; ↓: decrease; ↔: no significant change; *: statistically significant difference compared to the CG.

## Data Availability

The original contributions presented in the study are included in the article; further inquiries can be directed to the corresponding authors.
